# Heterogeneity within AML with CEBPA mutations; only CEBPA double mutations, but not single CEBPA mutations are associated with favourable prognosis

**DOI:** 10.1038/sj.bjc.6604977

**Published:** 2009-03-10

**Authors:** T Pabst, M Eyholzer, J Fos, B U Mueller

**Affiliations:** 1 Department of Medical Oncology and Clinical Research, University Hospital Bern and University Bern, Bern, Switzerland; 2 Department of Internal Medicine and Clinical Research, University Hospital Bern and University Bern, Bern, Switzerland

**Keywords:** prognosis, AML, mutations, *CEBPA*, risk assessment

## Abstract

CCAAT/enhancer binding protein alpha (*CEBPA)* mutations in AML are associated with favourable prognosis and are divided into N- and C-terminal mutations. The majority of AML patients have both types of mutations. We assessed the prognostic significance of single (*n*=7) and double (*n*=12) *CEBPA* mutations among 224 AML patients. Double *CEBPA* mutations conferred a decisively favourable overall (*P*=0.006) and disease-free survival (*P*=0.013). However, clinical outcome of patients with single *CEBPA* mutations was not different from CEBPA wild-type patients. In a multivariable analysis, only double – but not single – *CEBPA* mutations were identified as independent prognostic factors. These findings indicate heterogeneity within AML patients with *CEBPA* mutations.

One of the crucial transcription factors for myeloid cell development is the CCAAT/enhancer binding protein alpha (*CEBPA*) (Rosenbauer and Tenen, 2007). Targeted disruption of *CEBPA* results in a selective block of granulocyte maturation (Zhang *et al*, 1997), whereas conditional expression of *CEBPA* in precursor cells is sufficient to trigger granulocytic differentiation (Radomska *et al*, 1998). In AML patients, deregulation of *CEBPA* function is a common event comprising of genomic mutations (Pabst *et al*, 2001a; Frohling *et al*, 2004; Nerlov, 2004), transcriptional and post-transcriptional suppression (Pabst *et al*, 2001b; Helbling *et al*, 2004; Helbling *et al*, 2005), and inactivation by phosphorylation (Radomska *et al*, 2006).

Two types of *CEBPA* mutations are predominantly seen: frame-shift mutations in the N-terminal truncate the wild-type protein, whereas formation of a dominant-negative 30-kDa peptide initiated from an ATG further downstream is not affected. In contrast, C-terminal *CEBPA* mutations are in-frame insertions or deletions, thereby affecting DNA binding and homo- or heterodimerisation with other CEBP family members (Nerlov, 2004). The majority of AML patients with *CEBPA* mutations have both types of mutations, usually on different alleles. However, both types can occur as single *CEBPA* mutations.

Earlier study has indicated that *CEBPA* mutations in cytogenetically normal AML patients are associated with favourable prognosis (Frohling *et al*, 2004; Bienz *et al*, 2005; Gaidzik and Dohner (2008); Marcucci *et al*, 2008; Schlenk *et al*, 2008). Here, we assessed the prognostic significance of the different types of *CEBPA* mutations among a cohort of 224 consecutive AML patients of all subtypes. By direct sequencing, we identified 12 patients with double and 7 with single heterozygous *CEBPA* mutations. Patients with double *CEBPA* mutations represented the combination of C- and N-terminal mutation types. We found that favourable prognosis was exclusively associated with the double *CEBPA* mutation status, whereas the clinical outcome of patients with single *CEBPA* mutations did not differ from *CEBPA* wild-type patients.

## Materials and methods

### Patients

Malignant cells were collected at diagnosis from the Ficoll-separated mononucleated cells of bone marrow aspirates (132 patients) or peripheral blood (92 patients) from consecutive AML patients seen at the Department of Oncology, University Hospital, Bern, Switzerland between 2001 and 2007. Informed consent from all patients was obtained according to the Declaration of Helsinki, and the studies were approved by decisions of the local ethics committee of Bern, Switzerland. Patients were uniformly treated within the HOVON/SAKK 30/00 protocol.

### CEBPA mutational analysis

The entire coding region of the *CEBPA* gene was amplified using three overlapping PCR primer pairs as described earlier (Bienz *et al*, 2005). Sequences of the primers are listed in Supplementary Table S1. PCR products were sequenced in both directions. Abnormal sequencing results were repeated twice in both directions, including repetitions of PCR.

### Reporter gene assays

H1299 cells were transfected with 80 ng of luciferase plasmid encoding an oligomeric CEBPA site, together with 20 ng of pcDNA3-CEBPA or empty pcDNA3 vector along with 1.0 ng of CMV-Renilla plasmid. After 24 h, luciferase activities were determined (Pabst *et al*, 2001a). Each transfection experiment was repeated at least three times.

Protein isolation and western blot analyses: Fifty micrograms of extracted proteins from whole-cell lysates were analysed with a rabbit polyclonal antibody against CEBPA (1 : 1000; Santa Cruz Biotechnology, Santa Cruz, CA, USA.; sc-61).

### Statistical Analysis

Patients alive without progression or relapse by the time of analysis were censored at the time of their last follow-up. Time-to-event curves were constructed according to the Kaplan–Meier method and were compared with the log-rank *χ*^2^-test.

## Results and discussion

By sequencing the entire coding region, we identified 19 patients with *CEBPA* mutations in our cohort of 224 AML patients at diagnosis. Of these 12 patients had the combination of the N- and the C-terminal type of mutation, further referred here as double *CEBPA* mutation, and 7 patients had a single *CEBPA* mutation. Patients with the in-frame insertion polymorphism in the second transactivation domain (Wouters *et al*, 2007), with base pair variation(s) that did not lead to amino acid changes, or with in-frame sequence variations of unknown significance (one patient), were not considered in the further analysis. All *CEBPA* mutations are presented in detail in Supplementary Tables S2A and S2B. Two patients with a single point mutation encoding a novel stop codon located downstream of the alternative ATG at position 120 were not of the classic N-terminal mutation type, as formation of the 30-kDa peptide was also affected, but they were classified as having single *CEBPA* mutations because of the functional relevance of these particular mutations.

We identified *CEBPA* mutations exclusively in patients below the age of 61 years at diagnosis. Thus, only AML patients in this age range were studied in the control group of *CEBPA* wild-type patients, allowing comparison of patients treated within the same protocol. We detected no differences between patients with single and double *CEBPA* mutations with regard to FAB subtypes, leucocytes at diagnosis, percentage of peripheral blasts at diagnosis, bone marrow infiltration at diagnosis, and LDH levels (Supplementary Table S3).

Remarkably, additional molecular abnormalities were exclusively detected in the single *CEBPA* mutation group, including one patient with *FLT3-ITD* and one with *NPM1* mutation. All patients with *CEBPA* mutations had a normal karyotype with the exception of one patient with monosomy 7 in the single *CEBPA* mutation group and one with del6q24 in the double *CEBPA* mutation group. Results of karyotype and molecular analyses are given in Supplementary Table S4.

**Figure 1:**
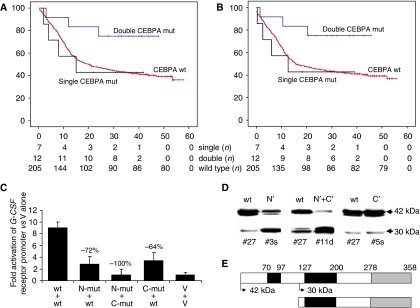
(**A**) Overall survival of AML patients without *CEBPA* mutations (wt; *n*=205), with a single (*n*=7), and with the combination of C- and N-terminal *CEBPA* mutations (double; *n*=12). Patients who are alive were censored at the last follow-up. *X*-axis indicates months, *Y*-axis is probability of survival. (**B**) Disease-free survival of AML patients without (wt; *n*=205)), with a single (*n*=7), or with double (*n*=12) *CEBPA* mutations. (**C**) Transient transfection experiments in H1299 cells using equal amounts of pcDNA3 expression plasmids encoding human *CEBPA* wild-type (wt), the N-terminal frame-shift mutation 245delC (as present in patient #3s in Supplementary Table S2), the C-terminal in-frame mutation 1079–1080insTCT (as present in patient #5s), and the combination of both plasmids. V: pcDNA3 expression plasmid alone. The luciferase reporter construct encodes an oligomeric *CEBPA* binding site. (**D**) Western blot analyses for *CEBPA* protein using whole-cell lysates of patients #27 (AML-M1 with a normal karyotype and no abnormalities in *CEBPA*, *FLT3*, and *NPM1*), #3s (AML-M2 with the N-terminal frame-shift mutation 245delC), #11d (AML-M1 with both the N-terminal 213insAG and the C-terminal 1088-1089insCCG mutations), and #5 s (AML-M1 with the C-terminal in-frame mutation 1079–1080insTCT). (**E**) Schematic presentation of *CEBPA* wild-type protein (upper panel) and the 30-kDa peptide initiated at the ATG at amino acid 120 (lower panel). Black bars indicate the two transactivation domains, and grey bars represent the region for DNA binding and homo-/heterodimerisation.

Comparing patients with single *vs* double *CEBPA* mutations, we found that the clinical outcome differed markedly (Supplementary Table S5). A complete remission after induction chemotherapy was achieved in all the 19 AML patients with *CEBPA* mutations. However, patients with single *CEBPA* mutations had a significantly worse median overall survival (OS) of 15 months (Figure 1A; *P*=0.006) and disease-free survival (DFS) of 12 months (Figure 1B; *P*=0.013) compared with patients with double *CEBPA* mutations in whom both median DFS and OS were not reached after a median follow-up of 34 months. Moreover, the course of the disease of patients with single *CEBPA* mutations was not different both for OS and DFS from *CEBPA* wild-type patients. Finally, in a multivariable analysis discriminating white blood cell count, age, *NPM1* mutations, and *FLT3-ITD*, only double *CEBPA* mutations, but not single *CEBPA* mutations, were identified as independent prognostic factors (Table 1). The *CEBPA* mutation status *per se* turned out to be of independent prognostic significance.

To illustrate the consequences of single *vs* double *CEBPA* mutations in terms of *CEBPA* function, we transfected H1299 cells with expression plasmids encoding *CEBPA* wild-type, an N-terminal frame-shift mutation, a C-terminal in-frame mutation, or the combination of both plasmids, and we determined the potential of these CEBPA peptides to activate a target promoter sequence as present in the G-CSF receptor promoter. As illustrated in Figure 1C, both the N- and the C-terminal *CEBPA* mutant peptides inhibited wild-type *CEBPA* protein in a dominant-negative manner, by reducing its activation potential by roughly 70%. However, it is important that the combination of both mutant constructs – in the absence of wild-type protein – failed to activate at all. This suggests that *CEBPA* activity is completely abolished in malignant cells of patients with double *CEBPA* mutations, whereas it is retained to some degree in patients with single *CEBPA* mutations. In addition, Figure 1D presents the various *CEBPA* peptides made in malignant cells from AML patients with different types of *CEBPA* mutations. It indicates that N-terminal frame-shift *CEBPA* mutations, in fact, decisively decrease the amount of wild-type *CEBPA* protein, whereas the 30-kDa peptide is detectable at a higher amount.

In conclusion, our findings indicate that there is relevant prognostic heterogeneity within AML patients with *CEBPA* mutations. Double *CEBPA* mutations are associated with distinctly favourable prognosis, whereas clinical outcome of AML patients with single *CEBPA* mutations is not different from *CEBPA* wild-type patients. However, the number of patients with a single *CEBPA* mutation is limited in our collection of patients, and larger series are needed to definitively assess its prognostic significance.

## Figures and Tables

**Table 1:** Multivariable analysis for overall survival and disease-free survival to assess the prognostic significance of single *vs* double *CEBPA* mutations in AML patients.

	**Overall survival hazard ratio (95%CI)**	***P*-value**	**Disease-free survival hazard ratio (95%CI)**	***P*-value**
CEBPA single^a^	1.23 (0.62–2.29)	0.52	1.56 (0.80–2.96)	0.28
CEBPA double^a^	0.28 (0.16–0.58)	<0.001*	0.30 (0.18–0.60)	<0.001*
NPM1^b^	0.56 (0.43–0.75)	<0.001*	0.54 (0.41–0.74)	<0.001*
FLT3 wt^c^	0.60 (0.25–0.77)	0.009*	0.62 (0.22–0.820)	0.022*
WBC^d^	1.38 (0.99–1.79)	0.022*	1.35 (0.98–1.74)	0.036*
Age^e^	1.15 (1.06–1.26)	0.008*	1.12 (1.02–1.18)	0.014*

Abbreviations: CI=confidence interval; *NPM1*=nucleophosmin; *FLT3*=*fms*-related tyrosine kinase 3; ITD=internal tandem duplication; WBC=white blood cell count; CEBPA=CCAAT/enhancer binding protein alpha; wt=wild-type.

a *CEBPA* mutation status was compared with *CEBPA* wild-type status.

b *NPM1* mutation status was compared with no *NPM1* mutation.

c *FLT3* wild-type status was compared with *FLT3-ITD* status.

d WBC higher than 20 × 10^9^l^–1^ were compared with lower than 20 × 10^9^ l^–1^.

e Age above 50 (to 60) years was compared with age below 50 years.

^*^P-values < 0.05 were considered significant.
