# Supplementary material for: Heterogeneity within AML with CEBPA mutations; only CEBPA double mutations, but not single CEBPA mutations are associated with favourable prognosis
Source: Br J Cancer. 2009 Mar 10;100(8):1343–6. doi: 10.1038/sj.bjc.6604977 (PMC2676545; doi:10.1038/sj.bjc.6604977)
Supplement: Supplementary Tables 1–5 [file 6604977x1.doc]

**Table S1: Sequences of the three PCR primer pairs A, B, and C applied to determine the *CEBPA* mutational status.**

| Gene | Nucleotide sequence |
| --- | --- |
|  |  |
| CEBPA-A-F | 5'-TCG CCA TGC CGG GAG AAC TCT AAC-3' |
| CEBPA-A-R | 5'-AGC TGC TTG GCT TCA TCC TCC T-3' |
| CEBPA-B-F | 5'-CCG CTG GTG ATC AAG CAG GA-3' |
| CEBPA-B-R | 5'-CCG GTA CTC GTT GCT GTT CT-3' |
| CEBPA-C-F | 5'-CAA GGC CAA GAA GTC GGT GGA CA-3' |
| CEBPA-C-R | 5'-CAC GGT CTG GGC AAG CCT CGA GAT-3' |

**Table S2A: Molecular characterization of *CEBPA* mutations.**

| # pat. | age | FAB | N-mutation C-mutation others | AA change |
| --- | --- | --- | --- | --- |
|  |  |  |  |  |
| Single CEBPA mutations: | | | | |
| 1 | 38 | M2 | 236-237insGC | A29fsX160 |
| 2 | 51 | M2 | 392-393insT | A91fsX107 |
| 3 | 55 | M2 | 245delG | G32fsX159 |
| 4 | 49 | M1 | 216-217insCG | P23fsX160 |
| 5 | 22 | M1 | 1079-1080insTCT | S310-311ins |
| 6* | 33 | M2 | 692C>G | Y181X |
| 7” | 53 | M1 | 1083C>T | Q312X |
|  |  |  |  |  |
| Double CEBPA mutations: | | | | |
| 1 | 40 | M2 | 563-564insCG | Y138fsX160 |
|  |  |  | 1094-1095insCTG | L315-316ins |
| 2 | 57 | M2 | 327-328insC | E59fsX107 |
|  |  |  | 1098-1099insGTC | V316-317ins |
| 3 | 52 | M2 | 395delC | F82fsX159 |
|  |  |  | 1076-1077insAAG | K309-310ins |
| 4 | 47 | M1 | 213delC | P22fsX159 |
|  |  |  | 1088-1089insTCT | S313-314ins |
| 5 | 44 | M2 | 327-328insC | E59fsX107 |
|  |  |  | 1098-1099insGTC | V316-317ins |
| 6 | 27 | M2 | 319insT | D106X |
|  |  |  | 940insAAG | 314insK |
| 7 | 40 | M1 | 291delC | A47fsX159 |
|  |  |  | 937insCAG | 313insQ |
| 8 | 52 | M2 | 286-287insTC | P46fsX160 |
|  |  |  | 1076-1077insCCG | K309-310ins |
| 9 | 51 | M2 | 327insT | E59fsX107 |
|  |  |  | 1094-1095insCTG | L315-316ins |
| 10 | 53 | M1 | 291insGC | A47fsX160 |
|  |  |  | 1076-1077insACG | K309-310ins |
| 11 | 39 | M1 | 213insAG | P22fsX160 |
|  |  |  | 1088-1089insCCG | S313-314ins |
| 12 | 29 | M1 | 395insCG | F82fsX160 |
|  |  |  | 937insCAG | 313insQ |

One patient had the following two point mutations of unknown significance and was not considered in this analysis: 1167G>A (inducing G340S) and 744-745GC>TT (inducing A199L). *This patient also had the following point mutations: 672C>G, 676C>T, 678-679GG>TT, and 683C>T; they were all located on the same allele also carrying the 692C>G (encoding a novel stop codon) as identified by allele specific cloning of PCR products. * and “: These two patients had point mutations encoding a novel stop codon downstream of the ATG located at amino acid position 120. Thus, formation of the wild-type 42kDa and of the 30ka peptides is both affected.

**Table S2B: Clinical course of AML patients with *CEBPA* mutations.**

| # pat. | Age | FAB | DFS OS follow-up relapse  (months) (months) (months) (months) | AA change |
| --- | --- | --- | --- | --- |
|  |  |  |  |  |
| Single CEBPA mutations: | | | | |
| 1 | 38 | M2 | 6 8 yes | A29fsX160 |
| 2 | 51 | M2 | 32 33 33 no | A91fsX107 |
| 3 | 55 | M2 | 12 15 yes | G32fsX159 |
| 4 | 49 | M1 | 1 3 yes | P23fsX160 |
| 5 | 22 | M1 | 22 23 23 no | S310-311ins |
| 6* | 33 | M2 | 42 43 43 no | Y181X |
| 7” | 53 | M1 | 3 5 yes | Q312X |
|  |  |  |  |  |
| Double CEBPA mutations: | | | | |
| 1 | 40 | M2 | 40 41 41 no | Y138fsX160 |
|  |  |  |  | L315-316ins |
| 2 | 57 | M2 | 23 28 28 no | E59fsX107 |
|  |  |  |  | V316-317ins |
| 3 | 52 | M2 | 2 3 yes | F82fsX159 |
|  |  |  |  | K309-310ins |
| 4 | 47 | M1 | 33 34 34 no | P22fsX159 |
|  |  |  |  | S313-314ins |
| 5 | 44 | M2 | 35 36 36 no | E59fsX107 |
|  |  |  |  | V316-317ins |
| 6 | 27 | M2 | 15 16 16 no | D106X |
|  |  |  |  | 314insK |
| 7 | 40 | M1 | 33 34 34 no | A47fsX159 |
|  |  |  |  | 313insQ |
| 8 | 52 | M2 | 28 32 32 no | P46fsX160 |
|  |  |  |  | K309-310ins |
| 9 | 51 | M2 | 11 12 yes | E59fsX107 |
|  |  |  |  | L315-316ins |
| 10 | 53 | M1 | 41 42 42 no | A47fsX160 |
|  |  |  |  | K309-310ins |
| 11 | 39 | M1 | 20 24 yes | P22fsX160 |
|  |  |  |  | S313-314ins |
| 12 | 29 | M1 | 37 38 38 no | F82fsX160 |
|  |  |  |  | 313insQ |

DFS: disease-free survival; OS: overall survival; Follow-up in months is indicated for patients alive and disease-free from time of diagnosis censored at their last follow-up visit.

**Table S3: Clinical characteristics of AML patients according to *CEBPA* mutational status.**

all CEBPA CEBPA CEBPA CEBPA

wt mut single double

(n=224) (n=205) (n=19) (n=7) (n=12)

­­­­­­­­­­­­­­­­­­­­­­­­­­­­­­­­­­­­­­­­­­_______________________________________________________________________

sex 102f/112m 93f/102 9f/10m 3f/4m 6f/6m

median age* 53 54 51 53 49

median WBC G/L 19.8 27.8 12.1 14.1 11.2

(range) (0.6-360) (0.6-360) (2.8-36) (3.2-36) (2.8-28)

median % blasts in blood 68 72 60 65 58

(range) (0-99) (0-99) (12-90) (22-90) (12-88)

median LDH units/L° 808 848 515 535 492

extramedullary

manifestations (n) 51 48 3 1 2

de novo AML, n 204 194 19 7 12

secondary AML, n 20 20 0 0 0

MDS (n) 17 17 0 0 0

therapy-related (n) 3 0 0 0 0

consolidation in CR1^+^

chemotherapy (n) 72 54 9 3 6

autologous transplant (n) 54 34 10 4 6

allogenous transplant (n) 33 33 0 0 0

FAB classification: M0 20 20 0 0 0

M1 61 53 8 3 5

M2 56 47 11 4 7

M4 48 48 0 0 0

M5 31 31 0 0 0

M6 5 5 0 0 0

M7 3 3 0 0 0

*All patients were younger than 61 years at diagnosis. °LDH normal <480 units/L. ^+^159 of 224 patients achieved a first complete remission after two cycles of induction chemotherapy and thus underwent consolidation therapy. P-values were calculated with the Mann-Whitney test. No significance of p<0.05 was achieved for any comparison between single versus double *CEBPA* mutation groups. Significant differences were observed between *CEBPA* mutant and *CEBPA* wild-type patients for WBC at diagnosis (p=0.012), for LDH at diagnosis (p=0.032), and for allogenous transplant as consolidation treatment in CR1 (p=0.003). Abbreviations: FAB, French American British classification; WBC, white blood cell count; LDH, lactate dehydrogenase. MDS, myelodysplastic syndrome.

**Table S4: Molecular and karyotype abnormalities of AML patients according to *CEBPA* mutational status.**

all CEBPA CEBPA CEBPA CEBPA

wt mut single double

(n=224) (n=205) (n=19) (n=7) (n=12)

­­­­­­­­­­­­­­­­­­­­­­­­­­­­­­­­­­­­­­­­­­_______________________________________________________________________

FLT3-ITD n(%) 63(28) 62 1 1 0

NPM1 mutation n(%) 102(46) 101 1 1 0

good-risk* n(%) 38(17) 38 0 0 0

intermediate-risk” n(%) 121(54) 104 17 6 11

bad-risk^+^ n(%) 65(29) 63 2 1° 1^#^

*Good-risk patients comprised t(8;21) and inv(16) with n=20, and n=18 patients, respectively. “Intermediate-risk patients showed a normal karyotype (n=111), +8 (n=8), and -Y (n=2). ^+^Bad-risk patients comprised all other karyotype results. °This patient had monosomy 7. ^#^This patient had del6q24. AML patients with *CEBPA* mutations had significantly less *FLT3*-*ITD* (p=0.023) and *NPM1* mutations (p=0.008).

**Table S5: Clinical outcome of AML patients according to *CEBPA* mutational status.**

all CEBPA CEBPA CEBPA CEBPA

wt mut single double

(n=224) (n=205) (n=19) (n=7) (n=12)

­­­­­­­­­­­­­­­­­­­­­­­­­­­­­­­­­­­­­­­­­­_______________________________________________________________________

CR1 achieved, n(%) 159(71) 140 19 7 12

Death in CR1, n 10 10 0 0 0

Relapse, n(%)

of patients with CR1 137(61) 130(63) 7(37) 4(54) 3(25)

OS at two years (%) 48 46 69 43 83

DFS at two years (%) 46 44 63 43 75

Patients disease-free

in follow-up, n(%) 83(37) 76 12 3 9

median, months 37 38 31 33 34

Abbreviations: first complete remission, CR1; overall survival, OS; disease-free survival, DFS. At two years, OS and DFS were significantly shorter in AML with single versus double *CEBPA* mutations (p=0.005, and p=0.011, respectively).
